# Cost and cost-effectiveness of Mohs micrographic surgery versus conventional excision for nonmelanoma skin cancer: A scoping review

**DOI:** 10.1016/j.jdin.2026.05.024

**Published:** 2026-06-06

**Authors:** Mehdi Boostani, Kimberly L. Brady, Michael J. Bax, Kegon T.K. Tan, Nathalie C. Zeitouni, Cerrene N. Giordano, Gyorgy Paragh

**Affiliations:** a Department of Dermatology, Roswell Park Comprehensive Cancer Center, Buffalo, New York; b Department of Economics, University at Buffalo, Buffalo, New York; c University of Arizona College of Medicine, Phoenix, Arizona

**Keywords:** basal cell carcinoma, cost, Mohs micrographic surgery, nonmelanoma skin cancer, squamous cell carcinoma, wide local excision

## Abstract

**Background:**

Nonmelanoma skin cancers (NMSCs) are commonly treated with conventional excision (CE) or Mohs micrographic surgery (MMS), but their relative cost-effectiveness remains uncertain.

**Objective:**

To summarize the available economic evidence comparing MMS and CE for NMSC and, secondarily, to identify methodological gaps that limit interpretation of comparative cost-effectiveness.

**Methods:**

We performed a scoping review of EMBASE, Scopus, PubMed, and Cochrane (January 11, 2025). Studies reporting costs and/or incremental cost-effectiveness between MMS and CE for NMSC were included.

**Results:**

Twelve studies (1999-2023) from the United States (*n* = 9), the Netherlands (*n* = 2), Australia (*n* = 1), and Iran (*n* = 1) were eligible. Reported costs ranged from $408-$3534 for MMS and $587-$2644 for CE, with directionality driven by repair complexity and care setting.

**Limitations:**

Main limitations included cross-system cost incomparability, predominance of observational designs with short horizons, inconsistent capture of downstream recurrence costs and patient-reported outcomes, and reliance on appropriate use criteria not designed for economic inference.

**Conclusion:**

Despite superior clinical margin control with MMS, inconsistent economic methods yield conflicting cost-effectiveness conclusions. Standardized, data-driven evaluations that incorporate comprehensive costs, long-term outcomes, and patient-centered measures are needed to guide the indication-specific use of MMS versus CE.

**Capsule Summary**

• This review evaluates economic studies comparing Mohs micrographic surgery and conventional excision, revealing heterogeneous methods and conflicting cost-effectiveness despite better margin control and lower recurrence with Mohs.
• For cost-conscious care, clinicians should consider the surgical setting, long-term recurrence risk, patient-perceived outcomes, and data on economic impact in indication-specific treatment selection.

## Introduction

Nonmelanoma skin cancers (NMSCs) are the most common malignancies worldwide.^1^ Treatment options vary widely in complexity, cost, and health care resource demands.^1^^,^^2^ Among surgical options, conventional excision (CE) and Mohs micrographic surgery (MMS) are the most widely used.^3^ MMS offers peripheral and deep *en face* margin assessment (PDEMA) and is associated with superior margin control and lower recurrence rates in many clinical scenarios.^4^ However, its use remains variable across health care systems.

Although MMS is generally favored for high-risk cases due to its clinical advantages,^5^ its relative cost-effectiveness compared to CE remains poorly defined. Numerous studies have attempted to evaluate this, but their findings are inconsistent. Many studies omit important factors such as long-term recurrence costs, patient-reported outcomes, and health system burden. A central analytic tool in this space is the incremental cost-effectiveness ratio (ICER).^6^ ICER is defined as the ratio of the difference in costs between 2 treatment strategies to the difference in their effectiveness, commonly expressed as cost per recurrence avoided or cost per quality-adjusted life year (QALY) gained. While ICER is intended to guide value-based decision-making, its accuracy and utility depend heavily on standardized methodology, comprehensive data inclusion, and a clearly defined analytical perspective.

Consequently, clinical decision-making is often guided by incomplete economic evidence. Current guidelines, including the appropriate use criteria (AUC), offer general recommendations but do not clearly define when MMS is unequivocally indicated.^7^ As a result, many cases fall into ambiguous categories, leading to variation in practice based on provider preference, access, and institutional norms.

This scoping review aims primarily to summarize the reported cost and cost-effectiveness findings comparing MMS and CE for NMSC, while also identifying the methodological gaps and reporting limitations that complicate interpretation and cross-study comparison. We outline the key data elements and structural improvements needed to build a high-quality evidence base that can inform more precise and economically sound surgical decisions in NMSC care.

## Materials and methods

We conducted a scoping review adhering to PRISMA-ScR^8^ and SWiM guidelines,^9^ searching EMBASE, Scopus, PubMed, and Cochrane on January 11, 2025. Economic methodology was appraised using the Joanna Briggs Institute checklist for economic evaluations,^10^ and the 2022 Consolidated Health Economic Evaluation Reporting Standards (CHEERS 2022) checklist.^11^ Full details of the search strategy, selection criteria, and quality assessment are provided in Supplementary Appendix 1, available via Mendeley at: https://data.mendeley.com/datasets/85njcy9m2r/2. Cost data were extracted as reported in the original studies and were not inflation-adjusted to a common price year. Values reported in currencies other than the US dollars were converted using the exchange rate for the year of publication.

## Results and discussion

### Study selection and characteristics

**Fig 1:**
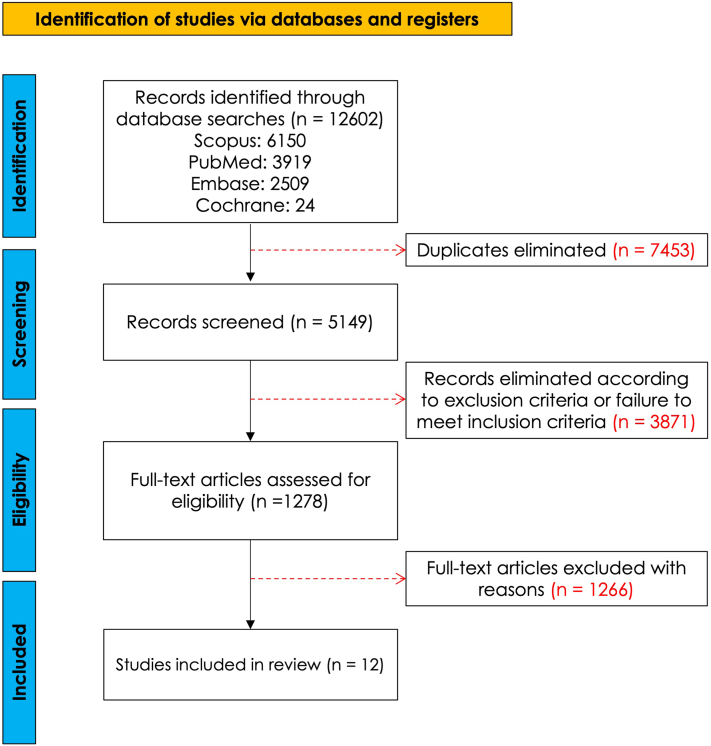
PRISMA flow diagram illustrating study selection process for scoping review. The flow diagram summarizes the identification, screening, eligibility assessment, and inclusion of studies in the scoping review. A total of 12,602 records were identified through database searches (Scopus: 6150; PubMed: 3919; Embase: 2509; Cochrane: 24). After removing 7453 duplicates, 5149 records were screened. Of these, 3871 were excluded for not meeting the inclusion criteria. The remaining 1278 full-text articles were assessed for eligibility, and 1266 were excluded with documented reasons. Ultimately, 12 studies were included in the review. This flow diagram adheres to the PRISMA guidelines. *PRISMA*, Preferred Reporting Items for Systematic Reviews and Meta-Analyses.

The systematic search identified a total of 12 studies published between 1999 and 2023 that met the inclusion criteria (Fig 1). These studies originated from the United States (9), Australia (1), the Netherlands (2), and Iran (1) and focused on the costs and cost-effectiveness of MMS versus CE for skin cancer treatments.

### Reported costs

The economic comparison between MMS and CE revealed mixed findings across health care settings, with reported episode costs ranging from $408 to $3534 for MMS and from $587 to $2644 for CE. These ranges represent reported study-level values rather than pooled summary estimates. Table I summarizes study characteristics, cost components, analytic approaches, and reported ICERs, while Table II presents comparator-specific incremental findings in a one-to-one format. Most studies were partial economic evaluations without ICERs.

**Table I:** Summary of the included studies

First author, y	Economic evaluation type (study classification)	Design (mutually exclusive)	Country	Objective/clinical context	Cost measures analyzed	ICER (denominator)	Conclusion (economic; outcome-specific)
Cook, 1998^12^	Cost description (partial economic evaluation)	Cost description/comparison study	United States	cf. the costs of MMS vs CE methods for NMSC	MMS and CE episode costs across settings and margin methods	Not applicable (no ICER)	Cost differences depend on comparator/setting; MMS is similar to office CE but less costly than ASC-based excision (cost-only; no ICER).
Bialy, 2004^3^	Cost comparison (partial economic evaluation)	Prospective cohort (cost comparison)	United States	Facial/auricular NMSC: cf. costs and margin adequacy of MMS vs CE	CPT/Medicare-based costs; included costs of additional procedures for inadequate margins	Not applicable (no ICER)	MMS may be economically favorable when the outcome is defined as margin adequacy/avoiding re-treatment; cost direction depends on CE margin method.
Essers, 2006^13^	Trial-based cost-effectiveness analysis (CEA)	Randomized controlled trial (trial-based CEA)	Netherlands	Facial BCC (primary and recurrent): assess cost-effectiveness of MMS vs CE	Total treatment costs; incremental analysis	€29,231 (primary) and €8094 (recurrent) per recurrence avoided	MMS is more costly but more effective (recurrence avoided); economically more favorable for recurrent BCC than primary under trial assumptions.
Mosterd, 2008^14^	Trial-based cost-effectiveness analysis (CEA)	Randomized controlled trial (5-y follow-up; trial-based CEA)	Netherlands	Primary vs recurrent facial BCC: CE vs MMS	Total costs and 5-y recurrence outcomes	€23,454 (primary) and €3171 (recurrent) per recurrence avoided	MMS is more costly but more effective; cost-effectiveness is more favorable for recurrent BCC (recurrence avoided) than primary.
Patel, 2010^15^	Model-based cost-effectiveness analysis (CEA)	Decision-analytic model (decision tree)	United States	BCC: modeled cost-effectiveness of MMS vs CE	Modeled costs using CPT/Medicare and recurrence costs	$24,173 per 1% recurrence avoided	MMS has a higher modeled cost; economic favorability depends on assumptions and willingness-to-pay for recurrence avoidance.
Ravitskiy, 2012^16^	Cost description/cost comparison (partial)	Cost description/comparison study	United States	cf. the costs of MMS vs CE with permanent or frozen margin control	Episode costs per tumor (different margin control strategies)	Not applicable (no ICER)	MMS has lower episode costs than CE comparators in this setting (cost-only; no incremental effectiveness metric).
Wilson, 2012^17^	Fee comparison (partial economic evaluation)	Retrospective cohort (fee comparison)	United States	cf. fees for ED&C vs CE vs MMS (including repair and follow-up)	Total fees: biopsy + procedure + repair + follow-up	Not applicable (no ICER)	MMS's higher fees are largely driven by repair costs; no cost-effectiveness inference without an incremental effectiveness metric.
Sebaratnam, 2016^18^	Cost comparison (partial; projected CE costs)	Prospective cohort (cost comparison)	Australia	BCC: true MMS costs vs projected CE costs for initial clearance	Mean episode costs	Not applicable (no ICER)	MMS slightly higher episode cost for initial clearance; cost-effectiveness not determined (no incremental metric).
Nassiripour, 2016^19^	Cost-effectiveness analysis (CEA; observational)	Retrospective cohort (CEA framework)	Iran	NMSC: cf. cost-effectiveness of MMS vs CE	Direct costs (surgery, hospitalization, meds, anesthesia, follow-up)	CE vs MMS: $263.22 per recurrence avoided (reported ICER)	MMS lower cost; effectiveness similar—conclusion depends on recurrence definition/ascertainment and setting.
Johnson, 2017^20^	Cost comparison (partial; setting comparison)	Retrospective case-matched comparison	United States	Outpatient vs OR treatment setting (MMS or CE)	Median costs by setting	Not applicable (no ICER)	Setting is a dominant cost driver; outpatient is substantially less costly than OR (not a MMS-CE effectiveness comparison).
Udkoff, 2022^21^	Model-based cost-utility analysis (CUA)	Decision-analytic model (Markov)	United States	T2a cSCC: MMS vs CE (WLE)	Direct costs including recurrence and repair pathways	Dominant (CE/WLE dominated by MMS; ICER not needed when dominant)	MMS is economically favorable in QALY-based analysis over 5 y (lower cost + higher QALYs).
Liu, 2023^22^	Cost comparison (partial; institutional cost analysis)	Retrospective chart review (cost comparison)	United States	Miami VA: MMS vs CE workflow costs	MMS + reconstruction vs CE + pathology	Not applicable (no ICER)	CE lower episode cost in this system; conclusion reflects institutional workflow/contracting (no incremental effectiveness metric).

This table summarizes the included studies comparing Mohs micrographic surgery (MMS) with conventional excision (CE) for nonmelanoma skin cancer, including study design, setting, objective/clinical context, cost measures analyzed, incremental cost-effectiveness ratio (ICER) where applicable, and the authors’ main economic conclusions. Studies were classified as partial economic evaluations (cost descriptions, cost analyses, or fee comparisons without incremental analysis) or full economic evaluations (incremental analyses reporting an ICER), including cost-effectiveness analyses (CEA) and cost-utility analyses (CUA). Because the evidence base is methodologically heterogeneous, this table is intended to provide a high-level overview of study characteristics and economic interpretations, rather than detailed one-to-one comparisons.

*ASC*, Ambulatory Surgical Center; *BCC*, basal cell carcinoma; *CE*, conventional excision; *CEA*, cost-effectiveness analysis; *CPT*, Current Procedural Terminology; *cSCC*, cutaneous squamous cell carcinoma; *CUA*, cost-utility analysis; *ED&C*, electrodesiccation and curettage; *ICER*, incremental cost-effectiveness ratio; *MMS*, Mohs micrographic surgery; *NMSC*, nonmelanoma skin cancer; *OR*, operating room; *QALY*, quality-adjusted life year; *VA*, Veterans Affairs; *WLE*, wide local excision.

**Table II:** Comparator-specific incremental cost and outcome comparisons for Mohs micrographic surgery versus comparator strategies

First author, y	Comparator	Incremental cost	Incremental outcome	Outcome metric
Cook, 1998^12^	Office-based CE with permanent-section margin assessment	+$76	Not directly compared	NR
	ASC-based CE with frozen-section margin assessment	−$730	Not directly compared	NR
Bialy, 2004^3^	CE with permanent-section margin assessment	+$106	Higher margin inadequacy with CE	Margin adequacy
	CE with frozen-section margin assessment	−$443	Higher margin inadequacy with CE	Margin adequacy
Essers, 2006^13^	CE (primary BCC)	+$318.6	Fewer recurrences with MMS	Recurrences avoided
	CE (recurrent BCC)	+$312.3	Fewer recurrences with MMS	Recurrences avoided
Mosterd, 2008^14^	CE (primary BCC)	+$377.5	Fewer recurrences with MMS	Recurrences avoided
	CE (recurrent BCC)	+$352.6	Greater recurrence reduction with MMS	Recurrences avoided
Patel, 2010^15^	Modeled CE	+$484	Lower recurrence with MMS	Recurrence avoided
Ravitskiy, 2012^16^	CE with permanent-section margin assessment	−$221	Not directly compared	NR
	CE with frozen-section margin assessment	−$395	Not directly compared	NR
	ASC-based CE with frozen-section margin assessment	−$1702	Not directly compared	NR
Wilson, 2012^17^	CE (total fees)	+$863	Fewer secondary procedures with MMS	Secondary procedures
Sebaratnam, 2016^18^	Projected CE (initial margin clearance)	+$41	Higher incomplete margins with CE	Incomplete margins
Nassiripour, 2016^19^	CE	−$210.6	Slightly lower nonrecurrence with MMS	Nonrecurrence
Johnson, 2017^20^	Operating room setting	−$9816	NR	NR
Udkoff, 2022^21^	CE/WLE	−$333.8	Higher effectiveness with MMS	QALY
Liu, 2023^22^	CE with pathology (VA workflow)	+$890.3	Not directly compared	NR

This table presents comparator-specific findings from the included studies in a one-to-one format to improve clarity when individual studies included more than 1 comparator or more than 1 comparison context. Each row represents a distinct comparison, with incremental cost reported as Mohs micrographic surgery (MMS) minus comparator. Incremental outcomes are presented separately from the corresponding outcome metrics to improve consistency and interpretability across rows. Outcome metrics vary by study and include recurrence avoidance, nonrecurrence, margin adequacy, secondary procedures, incomplete margins, and quality-adjusted life years (QALYs).

*ASC*, Ambulatory Surgical Center; *BCC*, basal cell carcinoma; *CE*, conventional excision; *MMS*, Mohs micrographic surgery; *NR*, not reported; *QALY*, quality-adjusted life year; *VA*, Veterans Affairs; *WLE*, wide local excision.

### Interpretation of economic and clinical findings

#### Clinical context and rationale for MMS

Conventional excision evaluates only a small fraction of the surgical margins.^23^ In contrast, PDEMA assesses the entire peripheral and deep margin, reducing false-negative margin assessment and enabling tissue-sparing surgery with lower recurrence risk.^24^^,^^25^ MMS is the most common PDEMA method in the United States,^25^ but its incremental benefit over CE varies by tumor and patient risk and must be weighed against longer procedure time and potentially higher upfront cost.

Currently, Mohs use is mainly guided by the Mohs AUC, which provides limited guidance by identifying certain NMSC cases as inappropriate for MMS, but leaves many scenarios ambiguously categorized as “uncertain,” subject to individual interpretation. Furthermore, AUC deems cases “appropriate” but does not provide clear guidelines for situations in which CE should be avoided, and only MMS or other forms of PDEMA should be considered. In the absence of definitive guidelines for triaging patients between MMS and CE, surgical decisions often depend on health care accessibility and referral practices rather than precise identification of patients who would benefit most from MMS's accuracy or CE's expediency and reduced health care utilization.

#### Variability in reported economic outcomes

Except for the Iranian study by Nassiripour *et al*^19^ all studies included in our review demonstrated superior clinical outcomes for MMS compared to CE. These outcomes included lower recurrence rates and better margin control, consistent with findings from previous studies that focused exclusively on clinical outcome differences between MMS and CE.^3^^,^^12^, ^13^, ^14^^,^^16^^,^^18^^,^^21^ Despite the almost uniform findings on clinical outcomes, the economic comparison between MMS and CE revealed mixed results with variability across healthcare settings and patient populations.

Several US studies demonstrated the favorable economics of MMS. A study by Cook and Zitelli. found that MMS costs were comparable to those of office-based CE ($1243 vs $1167) and substantially lower than those of CE performed in ambulatory surgical centers ($1973).^12^ Ravitskiy *et al* demonstrated that MMS was the least expensive option ($805 per tumor), outperforming both CE with permanent section margin control ($1026) and CE with frozen section margin control ($1200).^16^ Bialy *et al* showed that while MMS was slightly more expensive than CE with permanent sections ($937 vs $831), it cost markedly less than CE with frozen sections ($956 vs $1399).^3^

Wilson *et al* found that MMS was the most expensive option, primarily due to higher repair costs.^17^ Notably, repair costs for MMS averaged $735 per lesion, compared to $222 for excision, with a disproportionately higher use of complex closures such as flaps and grafts in the MMS group (125 MMS cases involved flaps or grafts vs only 1 excision case). While Liu *et al* demonstrated that in the Miami Veterans Affairs (VA) health care system, CE with pathology and reconstruction was significantly more cost-effective than MMS followed by VA-based reconstruction ($2643.85 vs $3534.12),^22^ this cost differential is primarily attributable to the institutional workflow. Specifically, MMS was performed offsite at a private facility with a flat contracted fee of $1,400, followed by reconstruction by VA plastic surgeons. In contrast, CE was performed entirely by VA plastic surgeons in a single operative setting. Although the study did not detail the types of repairs performed (eg, flap or graft) within each group, the cost difference appears to reflect this two-step care model and associated facility billing, rather than a standardized head-to-head single-setting comparison.

#### Influence of treatment setting and reconstruction patterns

The treatment setting emerged as a critical variable across several studies. Johnson *et al* highlighted a dramatic cost difference by setting, with MMS costing $1773 in the outpatient setting compared to $11,589 in the operating room.^20^ These findings suggest that in some cases, surgical setting selection may be more economically impactful than the choice of surgical technique. International studies were more variable. Sebaratnam *et al* reported MMS as slightly more expensive than CE in Australia ($628.47 vs $587.51).^18^ Notably, these studies did not report ICER, making it difficult to gauge the true economic impact of the increased cost when MMS offers superior clinical outcomes and theoretically would limit costs associated with recurrences.

#### Findings from incremental cost-effectiveness analyses

The studies that reported ICERs provided more rigorous economic evaluations. Udkoff *et al* concluded that MMS was both less costly and more effective than CE for high-risk cutaneous squamous cell carcinoma, saving $333.83 per patient while adding 0.043 QALY, with a 99.9% chance of being cost-effective.^21^ Nassiripour *et al* reported that MMS was less costly than CE in Iran ($408.1 vs $618.7) and found a nonrecurrence rate of 91.3% for MMS and 92.1% for CE. However, this small difference in effectiveness was not statistically significant, and the authors acknowledged potential limitations in the assessment of recurrence and selection bias related to the treatment setting. The resulting ICER of CE compared to MMS was $263.22 per recurrence avoided.^19^ Mosterd *et al* found that the ICER for MMS was $31,898 per recurrence avoided for primary basal cell carcinomas (BCCs) but dropped to $4312 for recurrent BCCs, making MMS cost-effective only in the recurrent setting.^14^ Essers *et al* found higher costs for MMS with ICERs of $36,831 and $10,198 per cancer recurrence avoided for primary and recurrent BCC, respectively.^13^ Patel *et al* calculated that MMS has an ICER of $24,173 per 1% recurrence avoided, raising questions about whether the added cost is justified in all cases.^15^

#### Sources of heterogeneity and methodological limitations

Variations in reimbursement models and resource availability can fundamentally alter economic calculations and treatment outcomes. The considerable variation in reported cost-effectiveness metrics largely stems from profound methodological differences across published studies, including substantial differences in cost measurement approaches, margin assessment techniques, the time horizons over which costs and outcomes were analyzed (eg, short-term vs long-term follow-up), and specific health care delivery settings evaluated.

Nevertheless, many of these studies did not adequately incorporate MMS's superior efficacy into their cost analyses, leading to an underestimation of its long-term value. Consequently, analyses focused on upfront costs may underestimate MMS’s long-term value.

Significant heterogeneity exists in cost reporting, with studies inconsistently including histopathological processing, repair procedures, facility fees, and follow-up care costs.^3^^,^^12^^,^^17^^,^^18^^,^^22^ Moreover, margin assessment methodologies varied substantially, ranging from theoretical margins to standard pathology to complete circumferential histological evaluation, which significantly impacted effectiveness calculations.^3^^,^^13^^,^^14^ Most studies focused on short-term outcomes and initial costs, systematically underestimating the long-term economic impacts of recurrences. The downstream costs of recurrences, including repeated surgeries, imaging, radiation therapy, immunotherapy, and reconstructive procedures, can dwarf initial treatment differences, and failing to account for them in the calculations can render conclusions invalid.^13^^,^^14^^,^^21^ Notably, dermoscopy by trained dermatologists can improve preoperative margin delineation, potentially reducing the number of stages.^26^ Yet none of the studies commented on the use of dermoscopy for margin delineation, a factor in margin assessment that can influence both the precision of excision and associated costs. High-risk BCC histologic subtypes, particularly sclerodermiform (morpheaform) and other infiltrative variants, are associated with greater subclinical extension and a higher risk of persistence/recurrence after standard excision, making them strong indications for MMS. However, the included economic studies did not consistently stratify results by histologic subtype, limiting interpretation and potentially diluting the value signal of MMS in these high-risk tumors. Furthermore, appropriate use of MMS should be based on preoperative histologic confirmation of BCC at the surgical site via prior biopsy (ideally in conjunction with dermoscopic mapping) to confirm the diagnosis and avoid artificially inflating apparent outcomes. Because the included studies did not consistently report biopsy confirmation or preoperative mapping workflows, variability in diagnostic pathways may contribute to heterogeneity and could bias comparisons of outcomes and costs. Selection bias was also common, as MMS was often reserved for more complex cases, where only 11 study addressed this selection bias directly using case-matching methodology.^20^ Patient-reported outcomes, particularly health-related quality-of-life measures, productivity impacts, and time lost from work, are essential for comprehensive economic evaluations as they capture the full value of health care interventions beyond clinical endpoints and enable standardized cost-utility analyses across different treatments and conditions.^27^ However, only 1 study in our review incorporated patient-centered outcomes (QALYs).^21^

#### Implications for treatment allocation and future research

The lack of high-quality evidence for clinical utility and cost-effectiveness is evident in how health care systems triage the use of MMS and CE. Currently, 4 formal guidelines or professional standards guide the appropriate use of MMS.^5^^,^^28^, ^29^, ^30^ Because there are no robust studies assessing the cost-effectiveness of NMSC surgery, cost-effectiveness considerations had only a limited impact on these criteria, and the criteria are mostly created to limit the use of Mohs in low-risk settings. Even more importantly, there is no guidance on when CE is inappropriate, given the significantly better outcomes of PDEMA and MMS.

Despite the limitations of prior comparative studies between CE and MMS (Table III), they provide a critical overview of the factors influencing the financial impact of these procedures. We summarized these key variables in Table IV, organized into 6 major categories of factors that influence cost-effectiveness: tumor-related factors, patient-related variables, financial and economic considerations, logistical and resource factors, clinical outcomes, and strategic factors. Because these sources of variation can dominate cost estimates, the apparent direction of findings (whether MMS appears more or less costly) often reflects setting and reconstruction patterns rather than the intrinsic economic value of the procedure, precluding a single generalizable cost-effectiveness conclusion for MMS versus CE across NMSC. Accordingly, the principal contribution of this review is to clarify why synthesis fails and to define what must be standardized to enable next-generation, decision-relevant evaluation. To address these gaps, we recommend establishing a comprehensive MMS-CE data portal to systematically capture consistent cost components and core clinical variables, including anatomic site risk, recurrence status, repair/closure patterns, diagnostic confirmation, histologic risk subtype, and dermoscopic or imaging-based margin assessment,^31^, ^32^, ^33^, ^34^, ^35^ alongside long-term recurrence pathways and patient-centered outcomes (including utilities/QALYs). Such standardization would also support an indication-driven allocation framework and improve comparability across countries with differing reimbursement, organizational structures, and care pathways.

**Table III:** Summary of financial and patient outcome limitations of included studies

First author, y	Limitations	
	Financial outcome limitations	Patient outcome limitations
Cook, 1998^12^	• Theoretical cost calculations rather than actual costs • Assumed uniform recurrence rates without patient-specific risk stratification • Excluded anesthesia, consultation, and immunopathology costs • Relied on 1996 reimbursement rates that may not reflect the true economic burden • No adjustment for regional cost variation or differing health care systems	• Limited follow-up for long-term recurrence assessment • No patient-reported outcomes, quality of life, or functional measures • Assumed reconstruction methods without patient preference or anatomic considerations • No cosmetic outcome or patient satisfaction evaluation
Bialy, 2004^3^	• Theoretical TSE costs rather than actual costs • Assumed all deep margins clear for TSE, potentially underestimating costs • Limited to Connecticut Medicare rates; may not generalize • Retreatment pathway assumptions may not reflect real practice • Sensitivity analysis showed results highly dependent on repair choice • No accounting for facility or regional cost variation	• Each patient served as their own control; not randomized • Possible bias: ENT surgeons are aware of tissue-area measurement (conservative margins) • Mohs surgeon, aware of cost focus, may have favored second-intention healing • High-positive margin rates may reflect specialty bias, not general TSE performance • No long-term recurrence, PROs, cosmetic, QoL, or healing time data • Limited to facial/auricular lesions, reducing generalizability
Essers, 2006^13^	• Hospital perspective only; excluded patient out-of-pocket, productivity, and societal costs • 18-30 mo follow-up may miss long-term recurrence costs • Single Dutch hospital costing; limited international generalizability • 2001 euro values may not reflect current economics • Indirect costs allocated as fixed 35% overhead — may misstate resource use • Bootstrap analysis showed 31% probability MMS inferior for primary BCC → substantial uncertainty	• High loss to follow-up (primary 16-22%, recurrent 7-9%) may bias recurrence estimates • Follow-up duration likely insufficient to capture 5+ year recurrences • QoL assessment limited to subset due to admin constraints/refusals • No cosmetic, functional, or satisfaction outcomes • 5-y recurrence extrapolated from expert opinion, not data • Mean substitution for missing cost data may bias results
Mosterd, 2008^14^	• Based on 2001 euros and Dutch costs, limited international applicability • 4% discount rate may not match current conditions • Mean substitution for missing cases in bootstrap analysis may bias results • Follow-up costs estimated as fixed outpatient visit costs (not measured) • Hospital perspective only; excluded travel/productivity/societal costs • ICERs are highly sensitive to small recurrence differences with wide CIs	• Potentially underpowered (lower recurrence, higher dropout: pBCC 23%, rBCC 27%) • Non-blinded assessments → detection bias • Standardized 3-mm margins for both arms may not reflect real practice • Recurrences can occur beyond 5 y; late recurrences acknowledged • Possible confounding from more aggressive BCCs in the MMS group (Cox model) • ITT included crossovers from SE to MMS, potentially biasing against SE • No patient-reported outcomes, QoL, or satisfaction measures
Patel, 2010^15^	• Decision-tree model with theoretical assumptions; no real patient data • Connecticut Medicare rates — limited generalizability • Deliberately minimized MMS costs and maximized SE costs → bias toward SE • Assumed uniform recurrence/retreatment without patient heterogeneity • Excluded facility fees, anesthesia, and many real-world variables • Based on 2008 CPT codes; may not reflect current practice/technology • Oversimplified reconstruction (all SE tissue rearrangement; MMS closure omitted)	• No actual patients; literature-derived recurrence rates • Assumed 67% second-stage rate for MMS regardless of tumor characteristics • No accounting for patient factors, comorbidities, preferences • No adjustments for surgeon experience, location, size, or histology • No QoL, functional outcomes, or satisfaction • Ignored long-term follow-up costs/late recurrences • No sensitivity analysis to test assumptions
Ravitskiy, 2012^16^	• Single practice (2 Mohs surgeons) — limited generalizability • Costs based on 2009 CMS fees for 11 region • Theoretical SSE costs with multiple assumptions (margins, same-day reconstruction) • Excluded immunopathology and general anesthesia costs (could favor SSE) • Assumed all recurrences treated with MMS and incomplete excisions re-excised with SSE • Excluded 5 tumors needing outside reconstruction	• Single-center; no randomized or matched comparison • SSE margin/reconstruction complexity based on experience, not empirical data • Excluding cases needing external reconstruction biases toward simpler cases • No long-term recurrence data from this cohort • High secondary-intention healing rate may reflect the surgeon's preference • No satisfaction, QoL, or functional outcomes • Could not evaluate actual SSE outcomes — theoretical only
Wilson, 2012^17^	• Single tertiary center; limited generalizability • Medicare fee-based costing vs actual costs • Missing lesion/margin data (3%/10%) required imputation • Assumed ≤5 tissue blocks per MMS stage without verification • Excluded recurrence-related costs • 2007 Medicare rules may be outdated • Decision-tree assumptions not fully empirical	• Retrospective observational design with selection bias (treatment chosen by biopsying clinician) • No randomized comparison • No cohort-specific recurrence outcomes (relied on literature) • Only 2-mo follow-up; misses long-term outcomes • No satisfaction, QoL, or functional outcomes • Tertiary-center patterns may not reflect community care • Tumor characteristics differed by treatment, complicating fair comparison
Sebaratnam, 2016^18^	• Australian MBS costing may not reflect true patient/societal costs • Excluded complications, income loss, or day-care admissions • Assumed subsequent CE costs equal initial procedure • Assumed all re-excisions achieve clear margins without further surgery • Results not translatable to other systems (esp. US) • MBS item numbers may not reflect actual resource use	• Open design with investigator aware of endpoints → bias • Compared true MMS costs with projected (not actual) CE outcomes • Tertiary referral center selection bias; complex BCCs → high incomplete-excision rate • Single investigator determined projected CE costs → systematic bias risk • Assumed adequate deep margins for all CE cases (unrealistic) • Bias toward CE by using the widest recommended margins and considering only radial margins • No actual recurrence, satisfaction, or QoL outcomes • Excluded cases needing plastic surgery reconstruction
Nassiripour, 2016^19^	• Governmental tariffs may not reflect actual costs or generalize internationally • USD conversion may not match purchasing power parity • Excluded nursing costs (assumed similar) • Excluded private hospital patients • Applied 2015 tariffs retrospectively to 2007-2010 data • Assumed all recurrences return to the same hospital • Fellowship vs specialist visit differences may inflate MMS costs • Medication cost differences may reflect specialty patterns, not necessity	• Non-randomized retrospective cohort with site-based selection bias • Recurrence is defined as >3 mo postsurgery → may miss early failures • Assumed nonreturn equals cure; may miss outside care • 96 patients excluded (death/loss to follow-up) → potential bias • No blinded outcome assessment • Significant associations (location, sex) with recurrence in the MMS group suggest confounding • Findings contradict multiple randomized studies favoring MMS
Johnson, 2017^20^	• Single center; OR costs vary widely by region/system • Small sample (18 matched pairs) → limited power/external validity • Used billing charges rather than actual costs • Retrospective design; unable to control surgeon preferences/practice patterns • Single region; costs may not represent national patterns • No long-term costs (recurrence, complications)	• Very small sample; limited generalizability • Most cases were melanoma, not NMSC • Gender mismatch between groups may introduce bias • Single-center experience may not reflect broader practice • Retrospective case-matched design is inferior to a randomized study • No clinical outcomes, recurrence, or satisfaction data • Limited tumor variety; no long-term comparative outcomes
Udkoff, 2022^21^	• Markov model from a single observational cohort, not RCT data • 2020 Medicare rates may not reflect actual/private costs • Assumed identical closure costs for MMS and WLE despite different defect sizes • Nodal-recurrence cost estimates based on limited data with wide variation • Payer perspective only; excluded patient time/travel/productivity • 5-y horizon may miss longer-term costs/benefits • National impact extrapolation assumes uniform practice patterns	• Underlying non-randomized cohort introduces bias • Group differences (more head/neck tumors in MMS) may favor WLE • Health utility values from the limited literature • Transition probabilities from single study, not a systematic synthesis • Assumed perfect compliance/follow-up • Monte Carlo simulations may not capture real-world variability • Pooled positive-margin rates may not reflect current practice • No accounting for surgeon experience/comorbidities/tumor factors
Liu, 2023^22^	• Single VA center; limited generalizability • OR cost/minute estimated from literature, not actual institutional data • Excluded costs for reoperation, complications, or recurrence • MMS flat fee ($1400) is a contract rate; may not reflect true costs • Pathology costs from Medicare reimbursements may not match resource use • Did not assess total episode-of-care or long-term costs	• Retrospective chart review with selection bias; no randomization/matching • No assessment of margin positivity, recurrence, or complications • Focused only on initial surgery; not subsequent procedures • No satisfaction, cosmetic, or QoL measures • Predominantly elderly male VA population (96% male) — limited generalizability • No long-term comparative effectiveness • TSE by plastic surgeons may not reflect typical dermatology practice • Could not control for tumor characteristics, complexity, or appropriateness

This table summarizes the primary financial and patient-outcome limitations reported in each study comparing Mohs micrographic surgery with conventional excision for nonmelanoma skin cancer. Limitations are categorized into economic and clinical domains.

*BCC*, Basal cell carcinoma; *CE*, conventional excision; *CMS*, Centers for Medicare & Medicaid Services; *CPT*, Current Procedural Terminology; *ENT*, ear, nose, and throat; *ICER*, incremental cost-effectiveness ratio; *ITT*, intention-to-treat; *MBS*, Medicare Benefits Schedule (Australia); *MMS*, Mohs micrographic surgery; *NMSC*, nonmelanoma skin cancer; *OR*, operating room; *PROs*, patient-reported outcomes; *QoL*, quality of life; *RCT*, randomized controlled trial; *rBCC*, recurrent basal cell carcinoma; *SE*, surgical excision; *SSE*, standard surgical excision; *TSE*, traditional surgical excision; *USD*, United States dollar; *VA*, Veterans Affairs; *WLE*, wide local excision.

**Table IV:** Comprehensive factors for optimizing treatment selection between MMS and CE for nonmelanoma skin cancers

Category	Factor	Description	Significance
Tumor-related	Tumor type	BCC, SCC, SCC in situ/Bowen's disease, other NMSCs	Different tumor types have varying recurrence risks and treatment responses
	Tumor location	Risk-stratified locations (H, M, L zones)	H-zone and M-zone (head and neck) locations typically benefit more from MMS
	Histological subtype	Aggressive (infiltrative, micronodular) vs indolent (nodular, superficial)	Aggressive subtypes are associated with higher recurrence rates with CE.
	Recurrence status	Primary vs recurrent	Recurrent tumors show much higher margin positivity
	Tumor size	Gradients (<1 cm, 1-2 cm, >2 cm)	Larger tumors of the same subtype have a higher recurrence with CE
	Perineural invasion	Present vs absent	Significantly increases recurrence risk with incomplete margins
	Clinical margin clarity	Distinct vs indistinct	Indistinct margins correlate with higher incomplete excision rates
	Previous treatment	Complete vs incomplete prior excision	Previously incompletely excised tumors benefit more from MMS
Patient-related	Immune status	Immunocompromised vs immunocompetent	Immunosuppression increases recurrence risk and mortality
	Genetic syndromes	Presence of predisposing conditions	Patients with Gorlin syndrome or similar conditions benefit more from MMS in certain instances
	Multiple tumors	Single vs multiple lesions	Same-day MMS for multiple tumors may offer a lower cost per tumor
Financial/economic	Treatment setting	Office vs ASC vs hospital OR	Office-based procedures cost significantly less than ASC and hospital OR
	Healthcare system	Country-specific reimbursement models	Significant variations across different countries
	Time horizon	Short-term vs 5+ year projections	Long-term analysis reveals MMS cost-effectiveness for high-risk tumors
	Comprehensive costs	Procedure, repairs, reconstruction costs	Repair complexity significantly impacts overall treatment cost
	Recurrence expenses	Downstream costs of managing recurrences	Advanced recurrences may require $100,000+ annually for treatment
Logistical/resource	MMS availability	Regional access to MMS-trained surgeons	Limited availability requires resource prioritization
	Institutional patterns	Academic vs private practice patterns	Influences case selection and complexity management
	Resource constraints	Budgetary and staffing limitations	May necessitate prioritization of highest-benefit cases
	Practice setting	Urban vs rural, tertiary vs primary care	Affects treatment options and follow-up capabilities
Clinical outcomes	Margin clearance	Expected complete vs incomplete rates	CE shows higher positive margin rates compared to MMS
	Recurrence rates	Projected 5-y recurrence likelihood	MMS shows lower rates of recurrence compared to CE
	Quality of life impact	QALYs gained or lost	There's QALY gain per high-risk SCC treated with MMS vs CE
	Additional procedures	Likelihood of needing re-excision	CE cases demonstrated a higher requirement for additional procedures in some studies
Strategic factors	Risk stratification	Methodology to prioritize high-benefit cases	Ensures MMS allocation to patients with the greatest potential benefit
	Cost-saving approach	Immediate vs long-term savings potential	Specific scenarios offer both immediate and long-term savings
	Comparative metrics	Value assessment within constraints	Enables systematic comparison of treatment value across cases

This table presents key factors identified for incorporation into a comprehensive calculator to optimize treatment decisions between MMS and CE for nonmelanoma skin cancers. The factors are categorized into tumor characteristics, patient factors, financial considerations, logistical realities, clinical outcomes, and strategic approaches. Implementation of these factors into a clinical decision tool would improve resource allocation and ensure patients receive the most appropriate treatment while maximizing health care efficiency.

*ASC*, Ambulatory Surgical Center; *BCC*, basal cell carcinoma; *CE*, conventional excision; *H, M, L zones*, high, medium, low risk anatomical zones; *MMS*, Mohs micrographic surgery; *NMSC*, nonmelanoma skin cancer; *OR*, operating room; *QALY*, quality-adjusted life year; *SCC*, squamous cell carcinoma.

#### Limitations

This review has several limitations. First, pooling studies across countries and systems (eg, the United States, VA, the Netherlands, and Australia) is problematic because reimbursement structures, case inclusion, and care pathways are not comparable. Moreover, the Mohs AUC were not designed for economic evaluation, so inferences about cost-effectiveness drawn from them are inherently limited. While we recommend a data-driven allocation framework, this review does not test or validate such a system, and acceptance of MMS as standard of care does not obviate the need for rigorous, evidence-based assessment of clinical indications alongside economic analysis. Mohs AUC was published in 2012 and likely changed case selection over time; therefore, earlier studies may reflect different utilization and anatomic distributions than current practice. Most included studies did not measure utilities/QALYs or patient-centered outcomes (eg, appearance/function and time-to-heal), which may underestimate the value of MMS for head and neck tumors when analyses focus mainly on short-term procedural costs. In addition, the evidence base was geographically concentrated, with most included studies originating from the United States, which may limit generalizability. Publication bias is also possible, particularly if studies with less clear or nonsignificant economic findings were less likely to be published.

### Study quality

Overall study quality was generally adequate, but substantial heterogeneity in cost-reporting methods, case characteristics, and reporting completeness limited generalizability and interpretation (Supplementary Tables I and II, available via Mendeley at https://data.mendeley.com/datasets/85njcy9m2r/2).

## Conclusion

The available evidence suggests that MMS generally offers better clinical outcomes than CE, but its relative cost-effectiveness remains uncertain because published studies yield conflicting results. Much of this inconsistency appears to reflect variation in repair complexity, surgical setting, reimbursement structure, analytic design, and the incomplete inclusion of long-term and patient-centered outcomes. Therefore, current data are insufficient to support a universally applicable economic preference for MMS or CE across nonmelanoma skin cancer. Instead, comparative value is likely to be indication- and setting-specific. Future studies should adopt longer time horizons, standardized cost reporting, clearly defined analytic perspectives, and consider the inclusion of patient-centered outcomes, such as QALYs, to improve comparability and decision relevance.

## Conflicts of interest

None disclosed.
